# In Vitro Evaluation of Mechanical, Surface, and Optical Properties of Restorative Materials Applied with Different Techniques

**DOI:** 10.3390/jfb15050128

**Published:** 2024-05-15

**Authors:** Merve Nezir, Suat Özcan

**Affiliations:** Department of Restorative Dentistry, Faculty of Dentistry, Gazi University, 06490 Ankara, Turkey; suatozcan@gazi.edu.tr

**Keywords:** compressive strength, glass ionomer, roughness, discoloration, resin composite

## Abstract

(1) Background: currently, the advantages of bulk-fill resin composite and high-viscosity glass ionomer materials have increased their use in dentistry; accordingly, their mechanical, surface, and optical properties have become more important. This study aimed to evaluate the mechanical, surface, and optical properties of three different restorative materials (a high-viscosity bulk-fill resin composite (TNC), a flowable bulk-fill resin composite (EBF), and a high-viscosity glass ionomer (FIX)) after application using different techniques (control, heat application, and ultrasonic activation). (2) Methods: specimens were prepared to assess the color stability and surface roughness (*n* = 12). The specimens were immersed in two different solutions for 14 days. For the compressive strength test, specimens were prepared using a Teflon mold (*n* = 12). (3) Results: among the specimens applied according to the manufacturer’s instructions and immersed in distilled water, it was observed that the surface roughness values of FIX on the 7th day were statistically significantly higher than the other materials (*p* < 0.05). The compressive strength values of EBF applied using ultrasonic activation were significantly lower than those of EBF applied using the other techniques (*p* < 0.05). (4) Conclusions: coffee can negatively affect the color stability of restorative materials, but discoloration may vary depending on differences in the content of the material. All materials evaluated in this study exhibited clinically acceptable surface roughness values. It can be concluded that flowable bulk-fill resin composite is the most durable material in terms of compressive strength, so it can be used in the restoration of posterior teeth, especially those exposed to intensive stress.

## 1. Introduction

Resin composites can be easily used as direct restorative materials in posterior teeth owing to their enhanced physical and aesthetic properties. They exhibit distinct surface and physical properties, which vary with changes in filler sizes and amounts. For this reason, several generations of resin composite restorative materials have emerged. The characteristics of these generations and restorative types must be understood to select the most appropriate restorative material for a given case [1].

Glass ionomer cements (GICs) were introduced to dentistry by Wilson and Kent in 1972. Their powder form is an acid-soluble calcium fluoroaluminosilicate glass similar to that of silicate but with a higher proportion of aluminosilicate that increases its reactivity with liquid [2]. High-viscosity GICs have been developed to increase the wear resistance of conventional GICs, strengthen their limited mechanical properties, and increase their indications limited to class one and five restorations. They are aimed to be an alternative to amalgam and resin composites as permanent restorative materials [3]. However, glass ionomers do not have sufficient physical and mechanical properties, which limits their use as permanent restorative materials in restorative dentistry. Nevertheless, a series of updates are being made to the structure and application method of glass ionomer materials to overcome such limitations. These updates include using heat application and ultrasonic activation during application to strengthen the material structure. Thermosetting is a relatively new technique that uses radiant heat to accelerate the setting reaction of conventional GICs. This technique helps overcome the problem of premature moisture sensitivity of GICs. Various researchers have investigated the effects of thermosetting on the mechanical properties of different GICs and reported an increase in surface microhardness and flexural strength [4]. Heat application has been noted to increase the mobility of both polymer segments and reactive free radicals formed during polymerization, which increases the degree of conversion of monomers into polymers and allows increased cross-linking of polymers [5].

Some of the advantages of applying the preheating technique to resin-containing materials in the literature include increasing the degree of conversion, improving the marginal adaptation of restorations by reducing viscosity, and reducing polymerization shrinkage. Applying heat to resin-containing materials increases the conversion rate of monomers to polymers and microhardness without accelerating the time at which maximum polymerization is achieved. This improvement is probably achieved through increased molecular mobility and collision frequency of reactive molecules [6]. In addition, Khan et al. reported that, after the application of ultrasonic activation to two different resin composite restorative materials for 60 s, the temperatures of the materials increased to 45–46 °C. The authors concluded that, as the temperature increases, the mobility of free radicals in the material also increases, consequently accelerating the polymerization reaction [7].

Dental restorations are exposed to complex loading scenarios during chewing, including pressure, tensile, and shear forces. Although tensile forces are noted as critical among these forces, compressive forces are also a cause for concern. Compressive strength is defined as the compressive strength of the compressed test specimen at the breaking point. Force is expressed not only as a measurement of atomic attraction or repulsion but also as a collective measurement of the interatomic forces of the compressed structure. It is known that the forces transmitted to restorations during chewing can break them or cause the tooth to fracture. Since most chewing forces are compressive, compressive strength has a particularly important role in the chewing process [8]. In addition to compressive strength tests, different tests can be used to assess the mechanical properties of restorative materials.

Surface roughness tests are among the tests frequently used to evaluate the mechanical properties of restorative materials [9]. In these tests, irregularities in the surface integrity of materials are determined. Such irregularities facilitate plaque retention, which can lead to gingival inflammation and caries formation, increase bacterial adhesion and superficial staining of restorative materials, and reduce the brightness of the restoration, causing its appearance to deteriorate and turn into an unaesthetic structure [10].

Apart from the mechanical properties of resin composite materials and GICs, their aesthetic properties are also of great importance. Restorative materials can absorb water and other liquids and pigments, resulting in discoloration. Particularly, discoloration of anterior restorations is considered an aesthetic failure and the restoration may need to be renewed. This means additional costs and time requirements for both the patient and the physician [11].

A limited number of studies in the literature have evaluated various optical, surface, and mechanical properties of resin composite materials and high-viscosity GICs after placement using different techniques [12,13,14,15,16,17,18,19]. This study aimed to evaluate the effects of applying different techniques on the optical, surface, and mechanical properties of three different restorative materials, which have recently begun to be used frequently in clinics owing to their advantages, such as advanced chemical and mechanical properties and ease of application. The first null hypothesis of this study was that there would be no difference in the surface roughness, color stability, and compressive strength of the restorative materials applied using the same techniques. The second null hypothesis was that the application technique used would not have any effect on the surface roughness, color stability, and compressive strength of the restorative materials. The third null hypothesis was that soaking in distilled water or coffee would not affect the surface roughness and color stability of the restorative materials.

## 2. Materials and Methods

**Figure 1:**
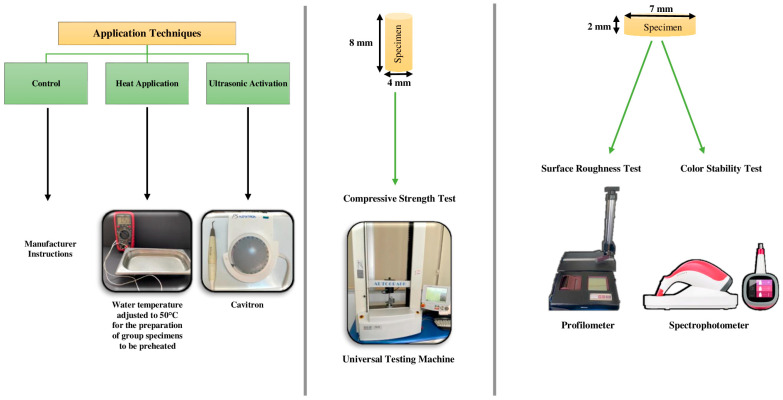
Study design.

The optical, surface, and mechanical properties of three different restorative materials, including a high-viscosity bulk-fill resin composite (Tetric N-Ceram Bulk-Fill, Ivoclar Vivadent, Schaan, Liechtenstein (TNC)), a flowable bulk-fill resin composite (Estelite Bulk-Fill Flow, Tokuyama Dental Corp., Tokyo, Japan (EBF)), and a high-viscosity GIC (Fuji IX GP, GC Corp., Tokyo, Japan (FIX)), were evaluated after heat application and ultrasonic activation. In control groups, the materials were applied in accordance with the manufacturer’s instructions. In heat application groups, the materials were immersed in water at 50 °C for 1 min (the temperature of the water was kept constant with the help of a thermocouple). In ultrasonic activation groups, during placement on the materials, a 5 s at 36 Hz ultrasonic activation was applied with a cavitron (VDW, Munich, Germany) (Figure 1). Ultrasonic energy is produced at the tip of the cavitron device and vibration occurs. The tip of the cavitron was placed inside the material while the material was being applied, and the generated ultrasonic energy was transmitted to the material. Ultrasonic activation application was conducted as follows: immediately after the material was placed in the molds, the ultrasonic tip of the cavitron was applied for 5 s by gentle movements from the center of the material in the mold to its periphery.

The materials used and their contents are shown in Table 1.

### 2.1. Color Stability and Surface Roughness Tests

**Figure 2:**
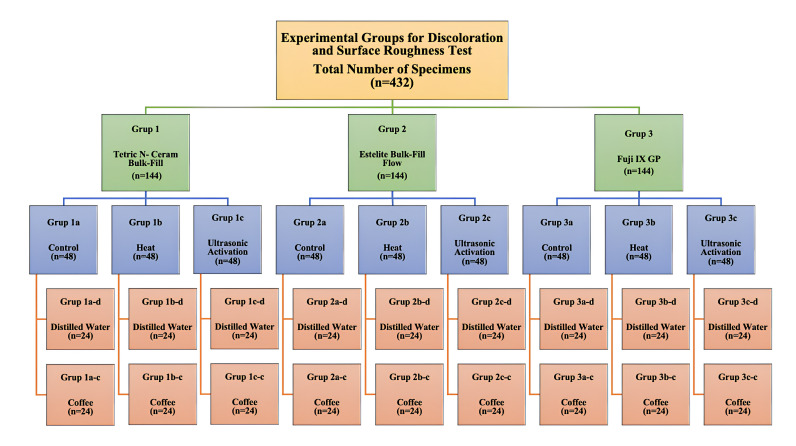
Experimental groups for color stability and surface roughness test.

Disk-shaped specimens (*n* = 12) with a diameter of 7 mm and a thickness of 2 mm were prepared from all three restorative materials for the color stability and surface roughness tests. Each restorative material was placed into Plexi-glass molds using different techniques, and the resin composite materials were polymerized using a light-emitting diode (LED) curing light device (D-Light Pro, GC Europe N.V., Leuven, Belgium) (High Power (HP) mode, light intensity: 1400 mW/cm^2^). The prepared specimens were kept in distilled water at 37 °C for 24 h. The surfaces of the specimens were polished using aluminum-oxide-coated discs (Figure 2).

Color measurements were conducted using a spectrophotometer (VITA Easyshade V Spectrophotometer, Vita Zahnfabrik, Bad Sackingen, Germany) to evaluate the optical properties of the materials. Discolorations were evaluated using the CIE L*a*b* system, where L* represents the brightness of the material on a scale from 0 (black) to 100 (white); a* represents hue and chroma on the red–green axis; and b* represents hue and chroma on the yellow–blue axis. The spectrophotometer was properly calibrated before each measurement following the manufacturer’s instructions. The difference between two colored specimens or two time periods is represented as ΔE*. Herein, ΔE* was calculated as follows:

ΔE* = [(ΔL*)^2^ + (Δa*)^2^ + (Δb*)^2^]^1/2^ (1)

Surface roughness was measured from three different directions using a surface-roughness-measuring device (Surftest SJ-301 Mitutoyo, IL, USA), and the average surface roughness was evaluated.

After initial measurements, the specimens were kept in two different solutions (distilled water (pH = 6.74) and coffee (Nescafe Classic, Nestle, Switzerland, 2 g of coffee powder was diffused in 200 mL of boiling water, pH = 5.66)) for 7 days, with a fresh solution prepared every day. After the coffee powder was dissolved in boiling water to simulate normal coffee consumption, the specimens were immediately immersed in the coffee. Thereafter, the measurements were repeated. After the measurements were completed, the surfaces of the specimens were examined with a stereomicroscope (Leica MZ 16, Leica Microsystems GmbH, Wetzlar, Germany) at ×40 magnification.

### 2.2. Compressive Strength Test

**Figure 3:**
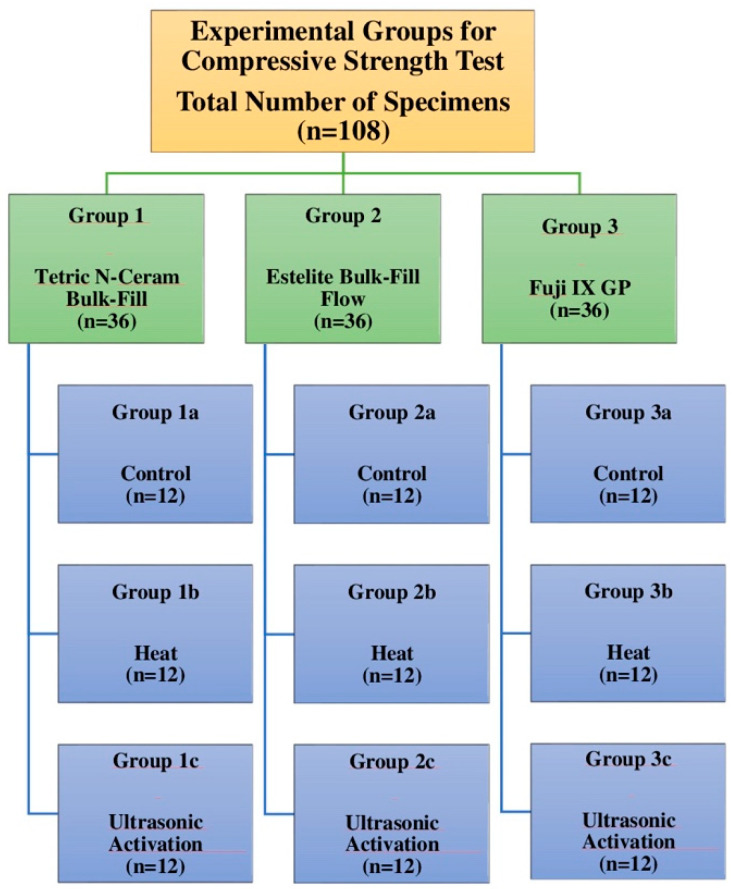
Experimental groups for compressive strength test.

A universal testing machine (Shimadzu IG-IS, Kyoto, Japan) was used to test the compressive strength of the materials. Specimens with a diameter of 4 mm and a height of 8 mm were prepared from all three materials using a Teflon mold (n = 12). While all parts of the Teflon mold were together, each restorative material was placed into cylinders formed using different techniques and the resin composite materials were polymerized using an LED curing light device (D-Light Pro, GC Europe N.V., Leuven, Belgium) (High Power (HP) mode, light intensity: 1400 mW/cm^2^). The upper surface of the last layer was covered with transparent tape and 1 mm thin microscope glass and constant pressure was applied. The pieces of the mold were carefully removed and cylindrical specimens with a diameter of 4 mm and a height of 8 mm were prepared (Figure 3). The prepared specimens were kept in distilled water at 37 °C for 24 h. All specimens were subjected to a compressive strength test on the universal testing machine. The specimens were placed vertically in contact with the center of the cylindrical steel tip of the universal testing machine. A continuously increasing force parallel to the long axis of the specimens was applied at a speed of 1 mm/min until fracture occurred. The force at which fracture occurred in each specimen was recorded in Newtons using a computer.

In the compressive strength test, the force (Newtons) applied at the moment fracture occurred was calculated as megapascals using the following formula:

Cs = 4F/πd^2^ (2)

Cs = compressive strength(3)

F = force at fracture (N)(4)

d = diameter of the specimen (mm)(5)

### 2.3. Statistical Analysis

IBM SPSS version 22 was used for the statistical analysis. The suitability of the parameters for normal distribution was evaluated using the Kolmogorov–Smirnov and Shapiro–Wilk tests; the evaluation revealed that the parameters had a normal distribution. Three-way ANOVA and Tukey’s test were used post hoc to evaluate the effects of the material, technique, and solution used on discoloration. Three-way repeated-measures ANOVA and post hoc Bonferroni test were conducted to determine the effects of the material, technique, and solution used on surface roughness. Conversely, two-way ANOVA and Tukey’s test were performed post hoc to assess the effects of the material and technique used on compressive strength. The significance level was set at *p* < 0.05.

## 3. Results

### 3.1. Color Stability

Among the materials applied according to the manufacturer’s instructions and kept in distilled water, the 7th- and 14th-day ∆E values of EBF were found to be significantly lower than those of the other materials (*p* < 0.05). Among the materials applied according to the manufacturer’s instructions and using heat and then kept in coffee, the 7th- and 14th-day ∆E values of TNC were significantly higher than those of the other materials (*p* < 0.05). The 7th- and 14th-day ∆E values of TNC applied using heat and kept in coffee were significantly higher than those of TNC applied using ultrasonic activation and kept in coffee (*p* < 0.05). In general, the discoloration values of the materials kept in coffee were higher than those of the materials kept in distilled water (Table 2 and Table 3).

### 3.2. Surface Roughness

**Figure 4:**
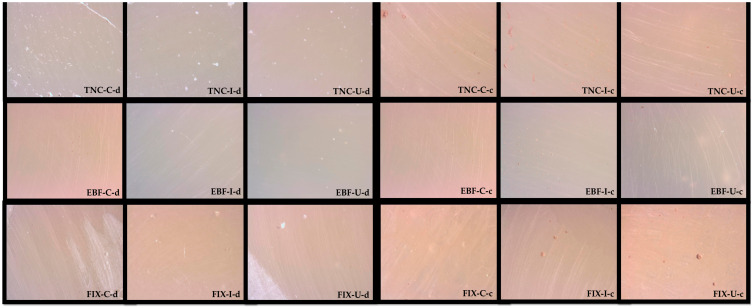
Stereomicroscope images of specimens kept in distilled water and coffee for 14 days. (TNC: Tetric N-Ceram Bulk-Fill, EBF: Estelite Bulk-Fill Flow, FIX: Fuji IX GP, C: control, H: heat, U: ultrasonic activation, d: distilled water, c: coffee).

Among the materials applied according to the manufacturer’s instructions and kept in distilled water, the 7th-day surface roughness values of FIX were found to be significantly higher than those of the other materials (*p* < 0.05). Among the materials applied using heat and kept in coffee, the 7th- and 14th-day surface roughness values of FIX were significantly higher than those of the other materials (*p* < 0.05). The 7th- and 14th-day surface roughness values of Group 3a–d were significantly higher than those of Group 3c and d (*p* < 0.05). The 14th-day surface roughness values of Group 1b–d were significantly higher than those of Group 1c and d (*p* < 0.05). The 7th-day surface roughness values of Group 2c and d were significantly higher than those of Group 2b–d (*p* < 0.05). In general, the surface roughness values of the materials kept in coffee were higher than those of the materials kept in distilled water (Figure 4, Table 4).

### 3.3. Compressive Strength

Among the materials applied according to the manufacturer’s instructions, the compressive strength values of EBF were significantly higher than those of the other materials (*p* < 0.05). The compressive strength values of EBF applied using ultrasonic activation were significantly lower than those of EBF applied using the other techniques (*p* < 0.05). Among the materials applied using ultrasonic activation, the compressive strength values of TNC were significantly higher than those of the other materials (*p* < 0.05). Among the materials applied using heat, the compressive strength values of FIX were significantly lower than those of the other materials (*p* < 0.05) (Table 5).

## 4. Discussion

Individuals’ expectations and awareness about dental aesthetics have been increasing in recent years. The success of restorative treatment depends not only on restoring function but also on correctly restoring tooth contour and aesthetics and maintaining stability of restoration color throughout the life of the restoration [20]. Color stability, which is defined as the ability of materials to resist discolorations, can be affected by environmental factors, the composition of materials, and technique-sensitive factors [21]. In addition, the color stability of restorative materials depends on the size of the resin matrix, size of the filler particles, type of coloring agents, and depth of polymerization [16]. The CIE L*a*b* system and a digital spectrophotometer are frequently used to evaluate the colorimetric qualities of dental materials objectively [22]. This approach reduces subjective color perception variability and provides consistency for identifying discolorations over time [23,24]. Therefore, in this study, the CIE L*a*b* system and a digital spectrophotometer were used to evaluate discoloration. According to the literature, there is a clinically visible discoloration when the ∆E value is 3.3 [25]. Paolone et al. [22] reported that there may be variability in the color stability of bulk-fill resin composite materials. They stated that this variability could be attributed to the material composition and the diversity of staining procedures. The present study found a visible discoloration in most groups. EBF exhibited the lowest discoloration values among the materials kept in distilled water. Conversely, TNC showed the highest discoloration values among the materials applied according to the manufacturer’s instructions and using heat and then kept in coffee. Therefore, the first null hypothesis of this study was rejected. Öztürk-Yeşilırmak et al. [26] evaluated the color stability of different restorative materials and reported that EBF showed less discoloration than did TNC. This result supports the present data. Other researchers have stated that the discoloration in materials depends on the amount of water absorption of the organic matrix they contain. The water absorption potential of Bis-GMA is greater than that of UDMA, TEGDMA, and Bis-EMA [27]. Additionally, Bis-GMA contains hydroxyl groups that are more sensitive to water absorption. TNC consists of an inorganic filler (57% by volume, 81.2% by weight), a 21% organic resin matrix, Bis-GMA, Bis-EMA, and UDMA [28]. EBF contains inorganic filler (56% by volume, 70% by weight), Bis-GMA, Bis-MPEPP, and TEGDMA [29,30]. Öztürk-Yeşilırmak et al. [26] reported that the organic matrix percentage by weight of EBF may be lower than that of TNC. Thus, the discoloration may be related to the organic matrix proportions. Although Bis-GMA is present in both materials, unlike TNC, EBF also contains a Bis-MPEPP monomer. Kawaguchi et al. reported that the viscosity of Bis-MPEPP monomers was lower than Bis-GMA [31]. One reason for the low viscosity of the EBF material may be the Bis-MPEPP it contains. In addition, Bis-MPEPP does not contain hydroxyl groups that contribute to water absorption. This may explain why EBF has less discoloration and TNC has more discoloration [26]. The color stability is directly related to the resin matrix of resin composites [32]. Kawaguchi et al. evaluated the mechanical and physical properties of Bis-MPEPP polymers. In the study, water absorption of Bis-MPEPP polymers and Bis-GMA were also evaluated. In addition, it has been reported that the water absorption values of Bis-GMA are higher than those of Bis-MPEPP. Researchers attributed this to Bis-GMA’s higher polarity and its inability to form a polymer with effective cross-linking due to the decrease in segmental mobility, which greatly affects the polymerization reaction [26]. In the present study, FIX showed less discoloration than did TNC when applied according to the manufacturer’s instructions and using heat. This outcome could be attributed to the application of a coating agent to the surface of FIX in accordance with the manufacturer’s recommendations. Çarıkçıoğlu [17] reported that the application of a coating agent to glass ionomer materials provided relatively good color stability. Other researchers have shown that the coating agent is more resistant to various forms of degradation under low-planning conditions [33]. In addition, GICs contain some water in their structure in the form of loosely bound water and tightly bound water, which is easily removed by dehydration. This feature may be effective in providing the material with higher resistance to discoloration by preventing additional water absorption from staining solutions [34].

In the present study, the discoloration values of TNC applied using heat and kept in coffee were higher than those of TNC applied using ultrasonic activation and kept in coffee. Therefore, the second null hypothesis of this study was rejected. The discoloration exhibited by a material has been shown to be either directly or indirectly related to the surface roughness of the material [35,36]. Chowdhury et al. [37] reported that, as the surface roughness increased, the discoloration also increased. In this study, the surface roughness values of TNC applied using heat and kept in coffee were higher than those of TNC applied using ultrasonic activation and kept in coffee (*p* = 0.119). The difference in the discoloration values could be explained by the increase in the surface roughness values of the materials.

According to the literature, the most commonly used solution for coloring is coffee [38]. In this study, coffee was used to color the restorative materials. The discoloration values of the materials kept in coffee were found to be higher than those of the materials kept in distilled water in most of the experimental groups. Therefore, the third null hypothesis of this study was rejected. Previous studies have reported that coffee is the most frequently applied solution for coloring resin composites [21,39,40,41,42]. Discoloration, which mostly occurs due to the adsorption and absorption of exogenous pigments, is an important and influential factor of the color instability of restorations [35]. Coffee solutions have yellow pigments of different polarities. Discoloration of resin composites exposed to coffee solutions may result from both adsorption and absorption of yellow pigments [43]. The yellow pigment molecules found in coffee are compatible with the polymer chains in the resin composite structure and, therefore, can be substantially effective in coloration [44].

The surface roughness of dental restorative materials can be affected by internal factors, such as differences in the size, volume, shape, and distribution of inorganic filler particles, as well as external factors, such as drugs and liquids to which materials are exposed. As the filler particle size of materials increases, the surface roughness also increases [45]. Bollen et al. [46] reported that Ra values above 0.2 µm may lead to a raised risk of periodontal inflammation and caries formation due to increased plaque accumulation. In the present study, the surface roughness values of all materials were below 0.2 µm. The highest surface roughness values were observed on the 7th day with FIX applied according to the manufacturer’s instructions and kept in distilled water. In addition, the surface roughness values of FIX applied using heat and kept in coffee were found to be significantly higher than those of the other materials. In restorative materials containing glass ionomers, the amount of the filler, size of the filler particles, and bond between the resin matrix and filler particles affect the surface roughness. Materials with large particles also have higher surface roughness values [47]. The high surface roughness values of FIX in the present study could be explained by the fact that the material had the largest filler particles. According to the literature, the kinetic energy transferred to materials by ultrasonic activation can increase the reaction conversion rate depending on the increase in temperature. In addition to increasing the temperature, ultrasonic activation can also contribute to the acceleration of the reaction by causing glass particles to come together and increasing the diffusion of reaction components. In this way, materials can be densified by reducing porosity and combining particles more tightly [48]. In this study, the surface roughness values of FIX applied according to the manufacturer’s instructions and kept in distilled water were found to be higher than those of FIX applied using ultrasonic activation and kept in distilled water. The results indicate that ultrasonic activation may have a positive effect on surface roughness by reducing porosity in materials.

Although the surface roughness of resin composite materials is related to the size and content of fillers, it is also affected by the filler particle type, silane bonding, and polymerization degree of polymer matrices [49]. However, ultrasonic activation can activate filler particles. Using vibration energy can cause changes in the filler/matrix distribution. Sonic excitation can eliminate the aggregation of particles and reduce porosity in the material structure [7]. The current study found that the surface roughness values of TNC applied using heat and kept in distilled water were higher on the 14th day than those of TNC applied using ultrasonic activation and kept in distilled water. Ultrasonic energy can reduce the porosity in the material structure and, consequently, the surface roughness. Conversely, the present study found that the 7th-day surface roughness values of EBF applied using ultrasonic activation and kept in distilled water were higher than those of EBF applied using heat and kept in distilled water. In their study, Khan et al. reported that more porosity occurred in flowable resin composites after ultrasonic activation was applied. This result could be attributed to the presence of structural porosity as a result of the production method of the material or technical difficulties during the placement of flowable resin composites [7]. In the study, ultrasonic activation was applied at a frequency of 15 Hz for 30 and 60 s with a cavitron device. However, there are studies in the literature that apply ultrasonic activation at different durations and frequencies with different cavitron devices [50,51,52]. Before starting this study, ultrasonic activation applications at different times and frequencies were tested on the materials. However, it was observed that long-term ultrasonic activation (30 and 60 s), especially on glass ionomer material, accelerated hardening considerably, so it was decided to apply 5 s as the most appropriate time. Differences in the results obtained in the studies may be due to differences in ultrasonic activation application times and frequencies. The fact that ultrasonic activation has an opposite effect on EBF compared with TNC in terms of surface roughness could be explained by the different production techniques of the materials or the different technical difficulty levels during placement. In the present study, the surface roughness values of the materials kept in coffee were higher than those of the other materials. Coffee solutions consist of water, and water absorption can deteriorate the structure of polymer-containing materials. When polymeric materials absorb water, they hydrolyze the binders and cause the chemical bond between the filler particles and the resin matrix to break down. The filler particles separate from the outer surface of materials, causing surface roughness [37]. Additionally, coffee may increase the degradation of the resin matrix of restorative materials owing to its high temperature [51]. In this study, after the coffee powder was dissolved in boiling water to simulate normal coffee consumption, the specimens were immediately immersed in the coffee [53,54].

Most chewing forces are compressive in nature [55]. Therefore, compressive strength is an essential parameter for restorative materials [56]. In the oral environment, restorations are exposed to occlusal stress. These forces act on the teeth and/or material, producing different reactions that lead to deformation, which can compromise their durability over time. This test is more suitable for comparing brittle materials that show relatively poor results under occlusal stresses [57]. It is a commonly used method to evaluate the strength of dental restorative materials [58]. Compressive strength test is an important method to evaluate the mechanical integrity of the material [59]. Since most masticatory forces fall into the category of compressive forces, it is of great importance to evaluate the durability of restorative materials in such situations. Compressive strength is one of the most important mechanical properties for posterior restorative materials and has an important role, especially in the chewing process [60]. Compressive strength tests reveal the critical value at which the restorative material breaks/fails during mastication. The minimum compressive strength value that can resist chewing forces in posterior teeth is 125 MPa [61]. Therefore, it is very important that the materials to be used in the restoration of teeth have sufficient compressive strength. For this reason, in this study, compressive strength test, which is an important parameter in the ability of materials to resist occlusal forces, was applied. The chemical composition of resin composites affects their mechanical properties [56]. Conversely, mechanical properties affect the long-term clinical success of materials. The properties of resin composites, such as durability and hardness, are directly related to the amount of filler [62]. However, the present study found that the compressive strength values of EBF were higher than those of the other materials applied according to the manufacturer’s instructions. While TNC had a higher filler ratio, it exhibited lower compressive strength values than did EBF. Therefore, values directly proportional to the filler amount and compressive strength values could not be obtained. Previous studies have reported that the compressive strength values of resin composites with the same filler content may not be the same depending on other factors such as polymerization type, polymerization shrinkage, transformation degree, and filler–matrix connection. In other words, compressive strength may be affected by the transformation degree, filler–matrix connection, and polymerization properties [62]. However, in this study, the compressive strength values of EBF applied using ultrasonic activation were lower than those of EBF applied using the other techniques. It has been reported in the literature that more porosity occurs in flowable resin composites after ultrasonic activation [7]. This porosity caused by ultrasonic activation in the material structure may negatively affect the compressive strength. In the present study, the compressive strength values of TNC were higher than those of the other materials applied using ultrasonic activation. The mechanical properties and clinical performance of resin composites are affected by different parameters. One of these parameters is the polymerization of resin composites. If resin composites are not polymerized effectively, unpolymerized or partially polymerized resin composite areas may remain at the base or between layers, decreasing durability [8]. Khan et al. [7] reported that the monomer conversion degree of high-viscosity bulk-fill resin composites increased after ultrasonic activation was applied. Raising the degree of monomer conversion means increasing polymerization. Increased polymerization may also positively affect the compressive strength of materials.

According to the literature, applying heat to glass ionomer materials increases the ion diffusion rate, accelerates the reaction, and reduces the working and setting time [6]. This study found that the compressive strength values of FIX were lower than those of the other materials applied using heat. Applying heat to glass ionomer materials can accelerate the hardening of the material, thus affecting the material structure and compressive strength.

A limitation of this study is that the spectrophotometer device used in this study (VITA Easyshade V, Vita Zahnfabrik, Bad Sackingen, Germany) is a clinical device (only working in “tooth mode”) and is generally not recommended for in vitro testing [63].

## 5. Conclusions

Within the limitations of this current study, it was concluded that:

- Coffee negatively affects the color stability of high-viscosity bulk-fill resin composites, flowable bulk-fill resin composites, and high-viscosity glass ionomers;
- High-viscosity bulk-fill resin composites, flowable bulk-fill resin composites, and high-viscosity glass ionomers exhibit clinically acceptable surface roughness. Nonetheless, individuals must be advised to be careful about the consumption of beverages that may increase surface roughness;
- Flowable bulk-fill resin composites exhibit the highest compressive strength values but applying ultrasonic activation to the material negatively affects the compressive strength.

## Figures and Tables

**Table 1:** Materials used in this study, contents, and application methods.

Material Name	Material Type	Manufacturer	Contents	Lot No.	Mean Particle Size	Method of Application
**Tetric N-Ceram Bulk-Fill**	High-Viscosity Bulk-Fill Composite Resin	Ivoclar Vivadent, Schaan, Liechtenstein	Filler content: 57% by volume, 81.2% by weight Barium glass, ytterbium trifluoride, mixed oxide, silicon dioxide, prepolymers Bis-GMA BisEMA, UDMA (camphorquinone)	W85057	1 µm	It was applied in a layer of 4 mm. It was polymerized with LED light device for 20 s.
**Estelite Bulk-Fill Flow**	Flowable Bulk-Fill Composite Resin	Tokuyama Dental Corp., Tokyo, Japan	Filler content: 56% by volume, 70% by weight New organic inorganic hybrid filler, supra nano spherical filler (SiO_2_-ZrOs), Bis-GMA, TEGDMA, BisMPEPP, Camphorquinone, Radical-Amplified Photopolymerization initiator	104E21	0.2 µm	It was applied in a layer of 4 mm. It was polymerized with LED light device for 10 s.
**Fuji IX GP**	High-Viscosity Glass Ionomer Restorative Material	GC Corp., Tokyo, Japan	Powder: Aluminosilicate glass, polyacrylic acid Liquid: Polyacrylic acid, water	210202A	13.43 µm	It was mixed in an amalgamator for 10 s.
**Equia Forte Coat**	Coating Agent	GC Corp., Tokyo, Japan	Methylmethacrylate, multifunctional methacrylate, camphorquinone	1906261	-	After being applied to the material surface, it was polymerized for 20 s with an LED light device.

Abbreviations: Bis-GMA, bisphenol A-glycidyl methacrylate; BisEMA, Ethoxylated bisphenol-A dimethacrylate; UDMA, Urethane dimethacrylate; TEGDMA, Triethylene glycol dimethacrylate; and Bis-MPEPP, 2,2-Bis[(4 methacryloxy polyethoxy) phenyl] propane.

**Table 2:** Evaluation of the effect of material, technique, and solution on color stability.

			∆E (7th Day)	∆E (14th Day)	∆E (7th Day–14th Day)
			Mean ± (Sd)	Mean ± (Sd)	Mean ± (Sd)
	**Group 1a** (TNC-C)		2.64 ± (1.4)	3.8 ± (1.45)	2.27 ± (1.74)
	**Group 1b** (TNC-H)		4.36 ± (1.79)	4.77 ± (2.1)	1.39 ± (1.22)
	**Group 1c** (TNC-U)		4.47 ± (2.86)	5.09 ± (3.29)	2.57 ± (1.66)
	**Group 2a** (EBF-C)		0.68 ± (0.38)	1.42 ± (0.68)	1.22 ± (0.74)
**Distilled Water**	**Group 2b** (EBF-H)		1.74 ± (2.59)	2.02 ± (2.31)	1.02 ± (0.38)
	**Group 2c** (EBF-U)		1.34 ± (0.57)	1.42 ± (0.48)	0.56 ± (0.26)
	**Group 3a** (FIX-C)		2.72 ± (1.77)	3.74 ± (1.77)	1.34 ± (0.66)
	**Group 3b** (FIX-H)		3.51 ± (2.24)	4.46 ± (2.41)	1.27 ± (0.74)
	**Group 3c** (FIX-U)		3.3 ± (1.84)	3.5 ± (1.58)	0.75 ± (0.31)
	**Group 1a** (TNC-C)		8.37 ± (1.29)	11.29 ± (1.83)	3.27 ± (0.97)
	**Group 1b** (TNC-H)		9.66 ± (1.95)	12.6 ± (1.95)	3.5 ± (0.91)
	**Group 1c** (TNC-U)		6.78 ± (1.33)	10.02 ± (1.64)	5.37 ± (1.63)
	**Group 2a** (EBF-C)		5.26 ± (1.29)	6.2 ± (2.34)	2.66 ± (1.71)
**Coffee**	**Group 2b** (EBF-H)		4.78 ± (0.99)	5.42 ± (1.37)	2.55 ± (0.71)
	**Group 2c** (EBF-U)		5.64 ± (2.95)	6.04 ± (2.79)	2.37 ± (0.94)
	**Group 3a** (FIX-C)		3.66 ± (2.18)	5.96 ± (1.59)	2.74 ± (1.09)
	**Group 3b** (FIX-H)		3.71 ± (2.31)	5.09 ± (1.2)	2.21 ± (1.04)
	**Group 3c** (FIX-U)		5.32 ± (4.76)	6.7 ± (5.11)	2.33 ± (1)
	*p* value for materials	C-d	0.002 *	0.001 *	0.102
		C-c	0.001 *	0.001 *	0.526
		H-d	0.041 *	0.023 *	0.623
		H-c	0.001 *	0.001 *	0.009 *
		U-d	0.006 *	0.003 *	0.001 *
		U-c	0.596	0.037 *	0.001 *
	*p* value for techniques	TNC-d	0.113	0.467	0.227
		TNC-c	0.001 *	0.013 *	0.001 *
		EBF-d	0.319	0.558	0.020 *
		EBF-c	0.618	0.720	0.860
		FIX-d	0.649	0.524	0.073
		FIX-c	0.450	0.532	0.513
	*p* value for solutions	TNC-C	0.001 *	0.001 *	0.131
		TNC-H	0.001 *	0.001 *	0.001 *
		TNC-U	0.033 *	0.001 *	0.001 *
		EBF-C	0.001 *	0.001 *	0.025 *
		EBF-H	0.003 *	0.001 *	0.001 *
		EBF-U	0.001 *	0.001 *	0.001 *
		FIX-C	0.304	0.008 *	0.003 *
		FIX-H	0.846	0.469	0.031 *
		FIX-U	0.226	0.075	0.001 *

Three-way ANOVA test: * *p* < 0.05 (C: control, H: heat, U: ultrasonic activation, d: distilled water, c: coffee).

**Table 3:** Mean L*, a*, and b* values of specimens in coffee groups.

		Initial			7th Day			14th Day	
	L	a	b	L	a	b	L	a	b
**Group 1a (TNC-C)**	80.93	−1.925	14.19	73.11	−0.53	16.29	70.14	−0.13	16.23
**Group 1b (TNC-H)**	79.82	−2.22	13.47	71.1	−0.4	16.07	67.81	−0.32	15.6
**Group 1c (TNC-U)**	77.34	−2.29	12.59	73.07	−0.32	16.96	68.08	−0.33	15.47
**Group 2a (EBF-C)**	74.87	−1.57	16.92	73.24	−0.19	21.42	72.43	0.04	21.44
**Group 2b (EBF-H)**	74.81	−1.82	17.22	72.97	−0.54	21.27	70.89	−0.36	20.36
**Group 2c (EBF-U)**	73.94	−2.11	15.72	71.57	−0.41	20.35	69.69	−0.43	19.59
**Group 3a (FIX-C)**	86.72	3.07	35.09	83.66	3.08	36.5	81.51	2.89	35.44
**Group 3b (FIX-H)**	86.98	3.04	35.45	84.03	2.96	36.67	82.42	2.79	35.7
**Group 3c (FIX-U)**	86.88	2.92	33.36	83.28	3.54	36.9	81.39	3.47	36.5

Abbreviations: TNC: Tetric N-Ceram Bulk-Fill, EBF: Estelite Bulk-Fill Flow, FIX: Fuji IX GP, C: control, H: heat, U: ultrasonic activation.

**Table 4:** Evaluation of the effect of material, technique, and solution on surface roughness change.

			Initial	7th Day	14th Day		T0-T1 *p*	T0-T2 *p*	T1-T2 *p*
	Roughness Values		Mean ± (Sd)	Mean ± (Sd)	Mean ± (Sd)	*p*			
	**Group 1a** (TNC-C)		0.086 ± (0.02)	0.099 ± (0.02)	0.116 ± (0.02)	0.067	0.053	0.063	0.645
	**Group 1b** (TNC-H)		0.111 ± (0.04)	0.106 ± (0.04)	0.142 ± (0.04)	0.184	0.896	0.306	0.205
	**Group 1c** (TNC-U)		0.087 ± (0.03)	0.091 ± (0.03)	0.095 ± (0.03)	0.739	1.000	1.000	1.000
	**Group 2a** (EBF-C)		0.104 ± (0.04)	0.095 ± (0.05)	0.104 ± (0.04)	0.452	0.613	1.000	1.000
**Distilled Water**	**Group 2b** (EBF-H)		0.101 ± (0.05)	0.07 ± (0.03)	0.089 ± (0.04)	0.001 *	0.001 *	1.000	0.482
	**Group 2c** (EBF-U)		0.106 ± (0.02)	0.112 ± (0.03)	0.112 ± (0.03)	0.585	0.890	1.000	1.000
	**Group 3a** (FIX-C)		0.15 ± (0.06)	0.157 ± (0.06)	0.162 ± (0.06)	0.753	1.000	1.000	1.000
	**Group 3b** (FIX-H)		0.122 ± (0.04)	0.122 ± (0.04)	0.126 ± (0.04)	0.982	1.000	1.000	1.000
	**Group 3c** (FIX-U)		0.139 ± (0.04)	0.1 ± (0.05)	0.113 ± (0.03)	0.001 *	0.001 *	0.049 *	0.934
	**Group 1a** (TNC-C)		0.102 ± (0.02)	0.106 ± (0.02)	0.125 ± (0.02)	0.131	0.928	0.135	0.274
	**Group 1b** (TNC-H)		0.097 ± (0.02)	0.116 ± (0.03)	0.124 ± (0.02)	0.001 *	0.004 *	0.043 *	1.000
	**Group 1c** (TNC-U)		0.104 ± (0.03)	0.112 ± (0.04)	0.103 ± (0.03)	0.347	0.411	1.000	1.000
	**Group 2a** (EBF-C)		0.103 ± (0.03)	0.134 ± (0.03)	0.147 ± (0.02)	0.001 *	0.001 *	0.005 *	0.810
Coffee	**Group 2b** (EBF-H)		0.103 ± (0.03)	0.12 ± (0.03)	0.122 ± (0.02)	0.001 *	0.001 *	0.042 *	1.000
	**Group 2c** (EBF-U)		0.119 ± (0.05)	0.138 ± (0.05)	0.136 ± (0.05)	0.005 *	0.002 *	0.056	1.000
	**Group 3a** (FIX-C)		0.156 ± (0.03)	0.152 ± (0.04)	0.173 ± (0.03)	0.289	1.000	0.338	0.486
	**Group 3b** (FIX-H)		0.122 ± (0.04)	0.151 ± (0.03)	0.167 ± (0.04)	0.004 *	0.002 *	0.029 *	0.630
	**Group 3c** (FIX-U)		0.151 ± (0.04)	0.166 ± (0.05)	0.186 ± (0.05)	0.004 *	0.009 *	0.006 *	0.704
	*p* value for materials	C-d	0.007 *	0.007 *	0.012 *				
		C-c	0.001 *	0.011 *	0.001 *				
		H-d	0.538	0.014 *	0.016 *				
		H-c	0.150	0.039 *	0.002 *				
		U-d	0.003 *	0.411	0.340				
		U-c	0.037 *	0.038 *	0.002 *				
	*p* value for techniques	TNC-d	0.132	0.557	0.007 *				
		TNC-c	0.783	0.766	0.119				
		EBF-d	0.957	0.042 *	0.370				
		EBF-c	0.513	0.558	0.295				
		FIX-d	0.402	0.048 *	0.045 *				
		FIX-c	0.098	0.635	0.566				
	*p* value for solutions	TNC-C	0.078	0.496	0.366				
		TNC-H	0.316	0.531	0.217				
		TNC-U	0.219	0.172	0.568				
		EBF-C	0.951	0.037 *	0.007 *				
		EBF-H	0.909	0.003 *	0.040 *				
		EBF-U	0.420	0.164	0.219				
		FIX-C	0.774	0.826	0.603				
		FIX-H	1.000	0.094	0.023 *				
		FIX-U	0.509	0.004 *	0.001 *				

Three-way repeated measures ANOVA test: * *p* < 0.05 (C: control, H: heat, U: ultrasonic activation, d: distilled water, c: coffee).

**Table 5:** Evaluation of compressive strength according to material and technique.

	C	H	U
	Mean ± (Sd)	Mean ± (Sd)	Mean ± (Sd)
**Group 1 (TNC)**	264.01 ± (57.38) ^a^	254.25 ± (43.54) ^a^	231.31 ± (40.46) ^a^
**Group 2 (EBF)**	328.91 ± (26.06) ^b^	288.69 ± (57.87) ^b^	98.67 ± (25.02) ^d^
**Group 3 (FIX)**	74.33 ± (19.48) ^c^	80.74 ± (21.69) ^c^	55.72 ± (18.99) ^e^

Two-way ANOVA test—the same superscript letters indicate no statistically significant differences (*p* < 0.05).

## Data Availability

The original contributions presented in the study are included in the article, further inquiries can be directed to the corresponding author.
